# On the Epidemic Mental Diseases of Children

**Published:** 1853-01-01

**Authors:** G. H. Cooke


### Original Communications.

---

### ON THE EPIDEMIC MENTAL DISEASES OF CHILDREN.

(*Translated from the German by G. H. Cooke, Esq.*)

We may be allowed, *in limine*, to reply to any objections which may be made against the application of the word epidemic to diseases which are purely and primarily mental.

We are too frequently compelled to use words borrowed from the physical world for the description of mental phenomena; to shrink from the employment of a new metaphor, merely because it does not express an exact resemblance.

It is well, however, that in each case we should remind ourselves that we are only using a metaphor, and that we should endeavour to ascertain and remember what are the chief points of contrast.

By neglect of this precaution, we may doubtless be misled by the words we use; an error than which none is more common, but which is not confined to the discussion of mental phenomena.

In the case before us, it is probable that the chief ground of hesitation to employ this epithet of pathological nomenclature lies in the fact, that in most cases of mental disease some consent of the will is necessary to their production.

The same objection, if it be admitted, would prohibit the employment of the words "disease," and the like, in reference to a large number of mental phenomena, which we regard as justly entitled to those epithets, and for which we should be at a loss to discover any other; for most of them might probably have been prevented by a strong exercise of the will.

But we cannot, as students of the mind in its pathological aspect, concede to it that freedom of the will which those who regard it from another point of view may claim for it. Our constant experience shows us, on the contrary, that it is in bondage to all external circumstances.

Of these external influences, the most powerful is, as might be anticipated, that of other wills, more powerful, either by position, or number, or in their own energy. This susceptibility, which we may call the impulse to imitate, is not at first an impediment to the will. In the infant, on the contrary, it is the first stimulus to voluntary movement. The first actions to which that name can be given are imitative of the motions and articulations of those about it.

"In the further development of the child, nature makes no sudden strides.

There is no abrupt line of demarcation between the earliest instinctive unconscious movements and the dawn of ideas, when the impressions made through the senses act on the sensorium.

"These ideas are necessarily at first obscure and undefined. For the reception of sensuous impressions there is required a preparation of the mind, which is not yet present, and is only slowly developed. This includes attention, comparison, and judgment.

"And there is no other inlet for ideas than through the nerves of sense and sensation. Spontaneous ideas are impossible to man under his existing organization.

"It is well known that children only learn to see and hear after a considerable time, though the organs of sight and hearing are from the first prepared to receive their appropriate impressions.

"There can be no question that the mental life of the child at this period is far inferior to the dreaming state of the adult: for though the suspended consciousness of the sleeper is scarcely stirred by the thread of ideas which flits across the mind, and is almost incapable of attention to them, yet these ideas may be perfectly clear in themselves, and even orderly; while in the child, when awake, they are only dim figures, without order and connexion.

"The faculty of speech is, as we know, acquired by imitation of the sounds heard.* The first modulated cries of the infant are not speech, but sounds without consciousness and significance; it learns to imitate the sounds presented to it, to show that it has received the impression they give. Gradually it associates ideas with the words, to which it then gives significance. It is not till a later period that there wakes up the higher faculty of recalling past ideas and the power of using words as the organ of thought."

It is still long before the child can attain that mental independence which is regarded as the characteristic of man; the most can hardly be said to attain it at all, but continue through life dependent on circumstances.

We constantly observe, in the amusements of children, that they are most absorbed in those which mimic the serious occupations of those around them—those of the school, the household, the family, the shop, and the church. Especially in times of popular excitement, we find the general topic repeated with perhaps greater earnestness, and the most engrossing plays of the children are the military attack or the parliamentary debate.

In all this we see nothing but what is natural; we are aware that it needs guidance and restraint, but this is easily applied—the need of rest and food, and the quiet discipline of home, separates the most ardent playmates, and subdues the most vivid enthusiasm.

That the faculty should be morbid in its manifestations, it is necessary that the general feeling of society should be unduly, or, at least, unusually excited, and that either the domestic tie be feeble, or the position of children in society misunderstood.

How widely spread, and how morbid an excitement may seize the youthful population under favouring influences is remarkably evinced in the children's crusade of the thirteenth century.

"At this period, the Holy Land had long been restored to the sway of the Saracens. Vexation for the loss, and an earnest desire for the recovery of this dearest possession of Christendom had spread with renewed earnestness among all the nations of the west. But the emissaries of Rome found no sympathy among the men, and not an arm was raised. They would not give their property and lives a useless sacrifice in the repeated effort at an achievement in which the skill and bravery of the past century had failed.

* In those cases in which the faculty of hearing is absent, speech is impossible till the mind is sufficiently exercised to imitate the motions of the mouth as observed by the eye.

"But the children's minds were kindled with brilliant dreams of the Holy Land, and of miraculous victories over the infidels, and it was impossible that some outburst of feeling could be long delayed.

"The first impulse was given by Etienne, a shepherd-boy of Cloies, near Vendôme, who must have possessed great address and talents. He gave himself out for an ambassador of Christ, who, he said, had appeared to him in a foreign garb, had received food at his hand, and given him a letter to the king. His sheep were said to have knelt down before him, to worship him, a miracle which perhaps was hardly needed to encircle him with the halo of sanctity. The shepherd-boys of the neighbourhood collected about him, and soon more than 30,000 persons streamed together to accept his revelations, and be transported by his preaching. He wrought miracles in St. Denys, the reports of which circulated with incredible rapidity through France. All rendered him homage, as the saint of the day and the messenger of God.

"The king, Philippe Auguste, alarmed at the excitement of so formidable a multitude, forbad the assemblies, with the sanction of the university of Paris. He might as well have forbidden an earthquake. Every day there started up new eight or ten-year old prophets, who preached, worked miracles, collected troops of young enthusiasts, and conducted them to the holy Etienne.

"To the inquiries put to these young pilgrims (for they were mostly clad in pilgrim's weeds) whither they were going, they replied, as with one voice: 'To God.' They went in orderly processions, headed with oriflammes; many carried wax candles, crosses, and censers, and they sang hymns without intermission, with most intense devotion, and to new melodies. In these hymns they often repeated the words 'Lord, raise up Christendom, and give us again the true cross.' It is to be regretted that the witnesses of a movement which thus ingulfed the whole of the youthful population, have not recorded either these hymns or the music to which they were sung. Even the few words which have been preserved have not come down to us in the vernacular dialect.

"It cannot be doubted that thus many of the fairest flowers of national poetry have been lost, however overwrought and morbid the excitement which produced them.*

"Many of the parents partook of the delusion, and furnished their children with arms and armour, or clad them in pilgrim's garb, and gave them a staff and wallet for their long journey. Some, who were kept back by force, wept day and night, and wasted away with fretting, or fell ill with nervous disorders, till they were allowed to go. Others made light of bolts and bars, and found means to elude the utmost vigilance of their attendants, that they might join the representatives of the holy Etienne, and at last behold that great crusade-preacher himself. All distinction of ranks was confounded: the children of counts and barons fled equally with those of burgesses and the lowest peasants. The richer parents, however, sent guides to accompany their children, many of whom it is probable were thus quietly rescued.

"Within a month from the commencement of the commotion, there was assembled at Vendôme an immense host of boys armed and unarmed, a few of them on horse, and among them not a few girls in male attire. The total number is reckoned at 30,000.

"They all acknowledged the beloved Etienne as their captain and guide to the Holy Land, which they purposed to rescue from the Saracens. They put him in a splendid chariot; the noblest of the youths, in splendid equestrian accoutrements, formed his body guard, which indeed he needed, to restrain the ardour of his followers, who blessed themselves if they could but get a

* Three or four years ago one of the Crusaders' hymns, with its melody, was discovered in Westphalia. It is characterized as far superior to any other music of that age which has descended to our times. It will be found in one of the numbers of *Evangelical Christendom* for 1850.

thread from his robe, when their devotion and enthusiasm had been inflamed by his preaching. On some of these occasions, a few were crushed to death in the violence of the press.

"In July, the extraordinary procession set out for Marseilles. It was hot and dry; but none of the difficulties of the pilgrimage, neither the drought on the hot and arid plains of Provence, nor the scarcity to which the poor must have been exposed after the first few days of the journey, could quench the ardour of their devotion and zeal. 'To Jerusalem!' was their cry, when they were asked of the astonished beholders whither they were going; and none doubted Etienne's promise that the sea should divide before them, and they should go to the holy land dry shod. Disappointed of this expectation, on their arrival at Marseilles they thankfully accepted the offer of two merchants of the city to convey them to Palestine without charge. They were still sufficiently numerous to fill seven large ships. Shortly after they set sail, two of the ships were wrecked in the bay, and not a soul saved. The remaining five were taken to Bugia and Alexandria, and all the children sold to the Saracens. It is satisfactory to know that the two merchants did not escape retributive justice. They were hanged in Sicily for another offence.

"At the same time a similar excitement sprang up in Germany, and repeated almost to the letter the incidents of the French boy crusade—the number of those drawn into it being probably somewhat larger. They were under two or more leaders, and went in two bodies toward the coast. It is probable that at least the half must have perished by the way, for the passes of the Alps were at that time very difficult, especially for such ill provided travellers; while the greater number of adults and women that attached themselves to these expeditions must have made the moral effects more disastrous.

One of the detachments reached Genoa in August; the other entered Italy by way of Lombardy. Their fate was various. A large number met the same fate as the children of Etienne's army, being kidnapped in the Italian sea-port towns, and sold into slavery; some entered into service in Italy; some fell victims to seduction or violence, and abandoned themselves to infamy; some of noble birth established themselves in patrician families in Genoa. A very few only returned home."

Hecker relates, on the testimony of a contemporary chronicler, a monk of Pirna, that twenty-five years later a similar movement seized the youthful population of Erfurt. Above a thousand of these assembled on a particular day, unknown to their parents, left the town together, and went dancing and leaping to Armstadt. They were fetched back the next day, but none of them could say who had enticed them, or wherefore they had gone. This appears to have been more connected with bodily indisposition than the boy crusades. Many of the children continued ill long afterwards with chorea and epilepsy. Hecker conjectures (I do not know on what ground) that the proximate cause of this event was the religious solemnities connected with the canonization of a landgravine of Thuringia. From its proximity in time to the former event, he very reasonably supposes an exciteable state of the "child world" at this epoch.

More than 200 years afterwards, in the year 1458, a time when the St. Vitus' dance was very prevalent, more than 100 children of Hale, in Suabia, set out, against the will of their parents, on a pilgrimage to Mont St. Michel, in Normandy. As all attempts to restrain them were fruitless, or hazardous, the corporation provided them a guide, and an ass to carry their baggage. They made the journey, offered their devotions to the archangel Michael, and returned.

All these mental epidemics (if so we may call them) had this in common, that they produced an impulse to bodily activity—an impulse which, especially in childhood, may be regarded as salutary and critical. I do not suppose (in defect of information on the subject) that any permanent effects were produced on those who survived the hardships of the journey. It is recorded that, on the contrary, those who were kept back suffered much with chorea and convulsions, as well as with the anguish of disappointment, and that some died in consequence.

There is another class in which we suspect the consequences may have been more lasting; more, we mean, which were connected with hallucinations, and which consisted in supposed commerce with the invisible world.

The belief of witchcraft prevailed throughout Europe from the time of the Reformation, or earlier, down to within a century of the present time. As I am now only concerned with so much of its history as relates to its influence on children, I have not here to decide how much was imposture or malignity, but set down all, with a merely fractional deduction, to the score of delusion.

It will be remembered that, in this unhappy superstition, the children were regarded as the chief victims, a delusion which, as we may anticipate, they almost universally confirmed.

"Nearly all the children were seized with the complaint. If their mothers were burned as witches, they themselves conversed with the devil, whom they saw hovering before them. They cried after their mothers, and all received answers from them. In sleep they felt themselves carried away by women in the form of cats, and when carried before the courts, they mentioned the names of the women who had thus carried them. This evidence, in which multitudes of children were unanimous, sufficed for the condemnation of those women to the stake. The children were carried into the churches, and were studiously kept awake. When overpowered by sleep, their dreams were repeated, and they described all the doing of the witches, the appearance of the devil, the food he set before them, the dances they had witnessed, the conversations and songs they had heard. When these witches were burnt, the evil was by no means stayed; the children soon found themselves similarly treated by other women. They all agreed in the affirmation that bolts and locks were of no avail to detain the witches from the observance of their Sabbath, and that they had themselves been cruelly scourged by witches, who were at the very time in prison. This was regarded by the judges as an additional motive to hasten the execution of the sentence, and even a girl of sixteen years was burnt on her confession of having carried three children to the witches' Sabbath.*

The convulsive disorders occasionally prevalent in schools and nunneries are too familiar to us, historically, to require that I should allude to the case treated by Boerhaave in the orphan asylum at Haarlem, or similar cases recorded in the treatises on hysterical affections.

From extensive inquiries which I have made among many of the great educational institutions in Great Britain, I cannot find any modern instances of these affections.

I do not know of any instance in which any demonstrations of juvenile ardour have taken place in defence of Mahometanism, nor can I find any records of a similar nature during the shorter history of Mormonism. To say nothing of the contrast of both these systems with the Christian system in their influence on the uncorrupted sentiments of youth, it is easy to perceive that neither the personal history of the Arabian prophet, nor that of his vulgar imitator, Joseph Smith, presents any thing adapted to win the affections and excite the enthusiasm of children, such as the history of our Lord presents.

* Though it does not strictly belong to my subject, I allude here to the prevalence of particular forms of mental affection at particular eras—chiefly to suggest to those interested in the inquiry a source of much interesting information. There is a department of the State-paper Office for a particular class of correspondence, which had long perplexed those who were engaged in deciphering the secret correspondence of the Stuart and revolutionary era. It was at last found that they were the letters of lunatics.

The same remark is applicable to ancient paganism, for the youthful processions in honour of some of its divinities were due to quite another sentiment; nor do any of our modern missionaries record anything similar in defence of their fading superstitions.

In some of the excitements of religious revivals at the close of the last century, the children took much part, and were evidently morbidly affected. I have not been able, unfortunately, to procure the exact information on this subject.*

It will be remembered, that a few years ago children were much pressed into the advocacy of the cause of total abstinence, much I believe to the moral injury of some, and with fatal results to others.

Beyond this, I do not know any modern events at all resembling those narrated at the beginning of this paper.†

If we are correct in attributing these phenomena to instincts of the youthful mind, we are compelled to inquire how it is that at a period so similar to the periods of the Crusades and the Reformation in the general religious and social excitement they have failed to recur.

I fear we shall hardly be justified in attributing it wholly to the more advanced civilization of our times, as offering to our youth patterns which they may safely imitate. Probably the true explanation is to be found in the more general knowledge of the nature of these excitements, and in the better understanding of the position of children in society. It is not improbable, that it is partly due to the increased longevity of modern times and the long peace of Europe, the numerical ratio of the youthful population to that of the adult and aged being thereby reduced, and their influence in society being proportionally diminished.

In some of the republics of central America, the low average of life through malaria and civil war has proved a fertile cause of incessant revolutions; their statesmen and magistrates being called to office almost in youth. Insanity and idiocy are also fearfully prevalent.

Creditable as this result may be to modern intelligence, and advantageous as it is to public decorum, I fear we have thus too entirely closed a safety valve, some action of which is advantageous to bodily welfare, and that the general health of the educated classes has suffered in consequence.

I do not refer to the supposed or actual increase of insanity at this period and in this country, which I think is not fully proved; nor to those severe affections of other parts of the nervous system, the prevalence of which in this country has compelled our physiologists on the researches which have given fame to this half century; but to what may almost be called the epidemic constitution of the educated classes of this country, and which constitutes the substratum

* Extract from a Letter by G. Lewis, Esq. "Wrexham, 7th April, 1851.—The principal fact I alluded to in connexion with the Welsh Revival Meetings, which I had observed on several occasions, was, that the excitement, after it had once commenced, could be kept up, and even renewed with considerable vigour when it had almost ceased, by mechanical means. Figure to yourself groups of young people from 12 to 20 years of age laying hold of each other by the hands, jumping and crying out for a considerable space of time, till their physical powers were nearly exhausted. Then some wicked wag, going behind them, pricking them in the elbows with pins or needles, which would, as a matter of course, make the one so pricked spring up, and his or her example would give fresh impetus to the whole circle; and it would be astonishing the length of time the jumping (*of all*) would go on again when apparently almost exhausted, when the impulse was once communicated by the example of one or two pricked. It was evidently the effect of example, or contagion, if you will; for the poor creatures were evidently at that time quite beyond the control of mental influences."

† It appears that in the "convulsive epidemic" in Cornwall, in 1814, the young were not predominantly affected.—See Mr. Cornish's account of it, *Med. and Phys. Journal*, vols. 31 and 32.

of those diseases, while it modifies the nature and in consequence the treatment of most others. I refer to the nervous asthenia so prevalent in both sexes, but chiefly in the female, during a few years, commencing from the establishment of puberty. In the majority of cases it passes away with the full development of the body, and may never during its continuance have amounted to disease—amounting only in its collective symptoms to what we habitually call delicacy of constitution, and which it is quite needless to describe.

It is impossible to collect statistics of what does not constitute an actual disease. I am compelled to rely on the statements of intelligent women, who almost unanimously testify in reply to my frequent inquiries, that this delicacy which they see in their daughters and granddaughters was almost unknown in their earlier days. This is confirmed by the testimony of the medical profession, that disease in general is more nervous and more asthenic than it was comparatively a short period ago. Probably the increased curability of diseases by nervous influence alone tends to further confirmation of the opinion.

It appears to me, that it is impossible to observe the symptoms of this condition, and the manner of its gradual abatement, without feeling that it is a result of exhaustion only to be relieved by a protracted, but not passive rest. It is, if I may use such a figure, a temporary old age, not very dissimilar to the climacteric disease of old age.

The present topic is one that chiefly concerns those who in any way have the care of the young—the clergy, preceptors, and parents. I am fully aware of the difficulty of the task at once to educate the child up to the point that shall qualify it to meet the excitement of the world, and to take care that the exercise thus given to the mind shall not restrain or impair the development of the body; and I am far from thinking that the difficulty is not on the whole judiciously met. I fear, however, that there is an undue tendency to call the young to aid of our religious institutions.

Far be it from me to discourage youthful decision and activity in religious matters, but to urge the necessity of care, in this more than in any other subject, that the child be not urged to do that which is properly the work of the man. If he does it in his childhood, there is a risk that he will not do it in manhood.*

Having hinted at what in some, not, I think, very many cases, is an error in the education of youth, I will mention, in conclusion, what seems to me the great desideratum of English society in cities. I mean scope and encouragement to active relaxation. It is with very little hope that they can be supplied that I allude to their needfulness. I fear that modern civilization has not as yet either the means or the wish to allow its younger servants that bodily exercise which might mitigate the strain which it imposes on their minds.

The proper mental and bodily relaxants are music and the dance. It is a subject of much congratulation that the former is now recognised as almost one of the necessaries of life. It is to be hoped that a better understanding of the value of the latter may ere long correct that mistaken morality which has driven it to late hours and heated drawing-rooms. No amusement is either healthy or moral which cannot be taken within the domestic circle, and at the hour when it is needed.

* Roddick, in his pamphlet on "Epidemic Democracy," blames the practice of teaching living languages to young children, believing that thus a premature expansion is given to their ideas and habits of thinking.

